# A Novel Experience Sampling Method Tool Integrating Momentary Assessments of Cognitive Biases: Two Compliance, Usability, and Measurement Reactivity Studies

**DOI:** 10.2196/32537

**Published:** 2022-03-28

**Authors:** Teresa Boemo, Angela Socastro, Ivan Blanco, Oscar Martin-Garcia, Ana Mar Pacheco-Romero, Raquel Rodríguez-Carvajal, Alvaro Sanchez-Lopez

**Affiliations:** 1 Complutense University of Madrid Madrid Spain; 2 Autonoma University of Madrid Madrid Spain

**Keywords:** experience sampling method, compliance, usability, measurement reactivity, emotion, cognitive biases

## Abstract

**Background:**

Experience sampling methods (ESMs) are increasingly being used to study ecological emotion dynamics in daily functioning through repeated assessments taken over several days. However, most of these ESM approaches are only based on self-report assessments, and therefore, studies on the ecological trajectories of their underlying mechanisms are scarce (ie, cognitive biases) and require evaluation through experimental tasks. We developed a novel ESM tool that integrates self-report measures of emotion and emotion regulation with a previously validated app-based cognitive task that allows for the assessment of underlying mechanisms during daily functioning.

**Objective:**

The objective of the study is to test this new tool and study its usability and the possible factors related to compliance with it in terms of latency and missing responses. Among the compliance predictors, we considered psychological and time-related variables, as well as usability, measurement reactivity, and participants’ satisfaction with the tool.

**Methods:**

We conducted 2 extensive ESM studies—study 1 (N=84; a total of 3 assessments per day for 5 days) and study 2 (N=135; a total of 3 assessments per day for 10 days).

**Results:**

In both studies, participants found the tool highly usable (average usability score >81). By using mixed regression models, we found both common and specific results for the compliance predictors. In both study 1 and study 2, latency was significantly predicted by the day (*P*<.001 and *P*=.003, respectively). Participants showed slower responses to the notification as the days of the study progressed. In study 2 but not in study 1, latency was further predicted by individual differences in overload with the use of the app, and missing responses were accounted for by individual differences in stress reactivity to notifications (*P*=.04). Thus, by using a more extensive design, participants who experienced higher overload during the study were characterized by slower responses to notifications (*P*=.01), whereas those who experienced higher stress reactivity to the notification system were characterized by higher missing responses.

**Conclusions:**

The new tool had high levels of usability. Furthermore, the study of compliance is of enormous importance when implementing novel ESM methods, including app-based cognitive tasks. The main predictors of latency and missing responses found across studies, specifically when using extensive ESM protocols (study 2), are methodology-related variables. Future research that integrates cognitive tasks in ESM designs should take these results into consideration by performing accurate estimations of participants’ response rates to facilitate the optimal quality of novel eHealth approaches, as in this study.

## Introduction

Mood and anxiety disorders are configured by a series of dysfunctional cognitive, emotional, and behavioral factors that occur over specific periods and are inherently dynamic: (1) they are influenced (predicted) by other thoughts, emotions, and behaviors preceding them, and (2) they also have consequences in the subsequent cognitions, moods, and behaviors of the individual. Although this dynamic interplay among psychological processes is supported by empirical research [1], and it was originally formulated by conceptual models guiding current treatments [2], this view has typically been ignored by standard diagnostic approaches. Diagnostic criteria (eg, Diagnostic and Statistical Manual of Mental Disorders, Fifth Edition [3]) are mainly focused on determining the existence of a given dysfunctional process for a given predefined period (eg, depressed mood during the past 2 weeks and generalized worry during the past 6 months), without considering their potential interplays with ongoing cognitive-affective processes that might be causing or maintaining the problems across time. The obvious consequence of ignoring time-varying data in this issue is a lack of knowledge on the proximal cognitive-affective mechanisms potentially driving the onset, maintenance, and recovery of affective dysfunctions [4].

In this line, to better understand the underlying mechanisms implicated in affective psychopathology, it is necessary to consider the ecological daily life dynamics of these cognitive-affective processes. Experience sampling methods (ESMs) and ecological momentary assessments [5] have emerged as crucial techniques to advance our knowledge on the dynamic psychological systems accounting for mental health and affective dysfunctions. They comprise repeated measures designs, where psychological assessments are performed several times a day for several days. The use of these methods has many advantages in addition to the rich and detailed information they provide. ESMs support in situ evaluations (state measures), which reduce the memory bias for self-reported retrospective assessments [6]. Furthermore, the clear improvement in the ecological validity of ESMs, in contrast to trait-based questionnaires, permits greater generalizability. It allows for the investigation of different individuals in their own contexts across time and situations, enriching theoretical and practical knowledge about the cognitive-affective processes of mental health and well-being [7]. To date, ESM research has clearly advanced the understanding of psychological processes involved in mental health, such as affective emotional reactivity [8], emotion regulation dynamics [9], the ecological use of simultaneous emotion regulation strategies [10], and the specific effectiveness of these strategies to regulate different momentary affective states [11]. However, these ESM measurements are still solely based on self-reports and do not allow for the evaluation of the underlying processes of these affective dynamics, namely cognitive-affective mechanisms. Individual preferences in the way that emotional information is attended to or interpreted (ie, cognitive biases) are typically assessed using experimental technologies such as eye tracking [12] in controlled laboratory conditions. These experimental methods allow for the capture of subtle mechanisms related to affective disorders [13] and the differential use of emotion regulation strategies [14-16] or even individual differences in psychological well-being [17]. However, to date, no research has fully integrated this type of cognitive bias assessment into ESM protocols, thus lacking a proper understanding of how their ecological momentary manifestations affect emotional experiences and their regulation in daily life.

Therefore, we developed a highly novel app system integrating a novel app-based cognitive task that allows for the measurement of attention and interpretation biases into a new ESM tool. This new method combines self-reported assessments of mood, stress appraisals, and emotion regulation use with momentary assessments of attention and interpretation biases during real-life functioning. Such assessments are based on the computerized version of the Scrambled Sentence Task (SST) [17], where participants are asked to freely unscramble a series of 6 scrambled word sentences displayed on the screen (eg, *born loser am I winner a*) using only 5 out of 6 words. The only 2 possible solutions to resolve the sentence are into a positive or a negative meaning (eg, *I am a born winner* or *I am a born loser*, respectively), and attentional processing of positive (eg, *winner*) and negative (eg, *loser*) are further assessed through advanced eye-tracking–based techniques [18]. In a series of experimental studies using this task, it was found that participants showing higher biases toward negative over positive information have poorer abilities to use emotion regulation strategies supporting negative affect downregulation (lower use of reappraisal and higher use of rumination) [19]. Furthermore, previous studies using the presented novel web-based app have demonstrated that ecological assessments of these negative cognitive biases assessed with this app-based cognitive task through mobile phones are predictive of poorer abilities to use emotion regulation strategies in their daily life functioning, ultimately leading to increased depressive and anxiety symptoms and reduced well-being in the face of major stressors [20]. Thus, given the large potential of these novel technical approaches to inform advanced health-related research and technology innovations, a thorough analysis of their usability and the conditions that facilitate their compliance is required. Ultimately, the aim of this study is to test the usability and feasibility of our method to be implemented for advanced research on the ecological mechanisms of mental health. Specifically, we first aim to study users’ perceived usability and satisfaction with the novel system, which integrates cognitive tasks within extensive ESM procedures. Second, we aim to establish the degree of measurement reactivity to the new method. Finally, we aim to establish factors that must be considered to maximize compliance with the use of the method, both in terms of latency and missing responses. For the latter, we exhaustively examine what factors are involved in effective compliance with this type of new ESM system, considering usability, satisfaction, reactivity, and emotional symptoms, as well as time-related variables as predictors.

Potential factors accounting for differences in compliance with the new ESM system were pre-established based on previously identified factors involved in the compliant use of ESM procedures in general [5,21,22]. Compliance with ESM protocols can be defined in two ways: (1) effectively answering the scheduled signals at the arranged times and (2) preventing the missing of assessment notifications. Therefore, it comprises time latencies and missing responses. Time latency is referred to as the time lag participants require to respond to a prompt or signal to start completing a momentary assessment, which ranges across studies from 90 seconds [23] to 3 hours [24]. Studying the latency of responses has several implications for the methodological quality of ESM studies and the quantity of available data, and it is closely related to missing rates.

Dealing with low compliance and abandonment rates has become a significant challenge when applying ESM designs in general. Owing to this, there is growing evidence exploring the possible systematic predictors of larger response latencies and missing data.

First, in terms of time-related variables, several studies have consistently found that missing rates tend to increase as ESM studies extend over time [25-30], although with some exceptions [31] (see available meta-analyses [29,32,33]). Furthermore, the effects of time of day (whether responding to ESM prompt signals during the morning, afternoon, or evening) are consistently related to missing responses [30,34,35]. For instance, some studies have found higher compliance rates in the afternoon (between 12 PM and 1:30 PM) and lower compliance rates in the morning (between 7:30 AM and 9 AM) [28]. Hence, in line with the revised literature, it is hypothesized that lower compliance with the new ESM system (increases in latency and missing responses) would emerge as the days of the study progress, as well as for earlier notifications during the day.

Second, there are relevant individual-related variables that should be considered when applying ESM procedures in general and specifically in ESM systems assessing mechanisms of mental health, such as the one proposed in this research. This refers to the consideration of how participants’ psychopathological conditions affect their compliance with ESM. This issue has been considered in previous research and has shown different results. Some studies have not found differences in ESM compliance among clinical conditions (schizophrenia, substance dependence, and anxiety disorders vs no diagnosis) [31]. However, other studies have found that higher missing rates are predicted by various clinical conditions, including anxiety and depression levels [29,30,32-35]. Therefore, the second aim of this study is to test whether individual differences in emotional symptoms (depression and anxiety levels) affect compliance with ESM. Considering that in this study, we evaluate 2 samples with subclinical symptom levels, it is hypothesized that no associations would be found between individual levels of depression and anxiety and compliance rates, supporting the feasibility of the ESM system for its use in this type of population.

Third, we considered it crucial to explore the implications that ESM approaches such as the one proposed have on individuals’ momentary experiences. Responding to ESM assessments at various times during the day entails paying regular attention to internal states and behaviors. Different results have been found regarding how the frequency of daily ESM assessments affects the dynamic trajectory of the psychological phenomenon being measured, thus generating different forms of measurement reactivity [36-38]. To date, few studies have evaluated the relationship between ESM compliance rates and experienced negative (burden) or positive (usefulness) measurement reactivity ESM. A recent study found an association between perceived burden and higher missing rates in longer ESM protocols [23]. According to this, to test the new ESM system developed for this study, we collected information regarding several measurement reactivity indicators that could be associated with different levels of compliance: experienced overload and stress generated by evaluation requirements, perceived usefulness of the assessment, and general satisfaction with the procedure. However, given the novelty of the topic under study, only the hypothesis regarding the relationship between burden and compliance is described. It is hypothesized that experiencing stress and overload would be related to lower compliance with the ESM protocol [23]. We further explore the relationship between positive reactivity variables, such as satisfaction and usefulness, and compliance rates during ESM because of the lack of previous literature on this topic.

Finally, when testing a new technological tool such as the one developed in this study, it is necessary to establish its degree of usability in terms of the system’s ease of use and learnability. Several studies have reported that patients with psychological disorders often find difficulties in engaging with technology that is challenging to use or that is perceived as irrelevant to their needs [39,40]. Therefore, given the purpose of this new ESM tool (ie, ultimately implementing it for use with clinical samples), we aim to initially test it in subclinical samples, considering the usability of the tool, analyzing its relationship with individual differences in depression and anxiety levels, and testing whether different individual levels of perceived usability have an influence on compliance with the novel ESM protocol. There is no previous evidence on this topic, despite its clear importance. Owing to this fact, no specific hypotheses were made about the relationship between usability and participants’ compliance with the new ESM approach.

To test these research issues, we conducted two studies testing the new app-based tool with different ESM regimes: The first study comprised a controlled experiment of 1 week (ie, study 1: a total of 5 days), and the second study extended the used ESM system to a larger period, comparable with regimes used in other types of ESM studies (ie, study 2: a total of 10 days), as described in the following sections.

## Study 1

### Methods

#### Participants

A sample of 84 undergraduate students (age: mean 20.05, SD 2.19 years) was recruited from the Faculties of Psychology of Complutense University of Madrid and the Autonomous University of Madrid. The participants received extra credit for participating in the study.

#### Procedure

Participants attended an introduction session in groups of 15 to 20, during which they received information about the study protocol and completed the informed consent forms, as well as baseline questionnaires assessing demographic variables and depressive and anxiety symptom levels. During the introductory session, participants downloaded the ESM tool, comprising a mobile app, and performed practice trials to become familiar with its use. A day after the introduction session, participants were instructed to complete the ESM assessment each time they received a new signal notification. A systematic sampling approach was used to determine random signaling schedules. Experience sampling assessments were programmed to be sent to participants 3 times a day for 5 days. These assessments were prompted randomly between 9 AM and 9 PM at three time intervals (9 AM to 1 PM, 1 PM to 5 PM, and 5 PM to 9 PM). Participants had 1 hour since they received the notification to complete the assessment. In each ESM assessment, they completed measures of stress, current affect, and use of emotion regulation strategies and performed a cognitive bias task. Furthermore, using the software, we generated a database where we gathered compliance-related information, such as the latency of response, missing assessments, and abandonment rates. At the end of the study, participants completed a brief questionnaire that accounted for variables related to measurement reactivity (app stress, app overload, and app usefulness) and user experience (usability and satisfaction). Questionnaires at baseline and the end of the study were gathered using Qualtrics (Qualtrics International Inc) software.

#### Instruments

##### Baseline Measures

In the initial introduction session, participants rated their levels of depression and anxiety, which were measured with the Center for Epidemiologic Studies–Depression (CES-D 8 [41]) scale and the Generalized Anxiety Disorder-7 (GAD-7 [42]) scale, respectively.

The CES-D 8 [41] is a screening scale used to evaluate depressive symptom levels in the past week. It contains 8 items that evaluate the severity of symptoms, which show good reliability in both general and clinically depressed samples [43,44]. The internal consistency in this study was *α*=.86.

GAD-7 [42] is a 7-item screening questionnaire that evaluates the severity of anxiety symptom levels comprising emotional and cognitive symptoms of anxiety. This measure has good reliability and validity in both general and clinically anxious samples [45,46]. The internal consistency in this study was *α*=.89.

##### ESM Assessments

The app comprised momentary self-reported assessments of several psychological states, including self-reports of perceived ongoing stress, use of emotion regulation strategies in response to ongoing stress, motivational factors, and current mood states*.*

Participants also completed a novel app-based cognitive task assessing momentary attention and interpretation biases in each survey. We created a computerized SST [17] that allows for app-based assessments of these cognitive biases. This task has been previously validated for the evaluation of attention and interpretation bias in various formats, such as computers [18], and it has already been used in mobile phones for implementation in ESM procedures [20]. Participants were required to complete 20 sentences at each ESM assessment a total of 3 times a day for 5 days. The full details of the ESM assessments in the new tool are provided in Multimedia Appendix 1.

##### Assessments at the End of the Study

Once participants completed the ESM part of the study, they received a final questionnaire to measure variables related to the use of the app: usability, satisfaction, app stress, app overload, and app usefulness. They completed these questionnaires using Qualtrics. To date, there are no validated evaluation protocols to assess these characteristics for eHealth mobile apps. Therefore, for some of the variables of interest, we opted to select single items from the Mobile Application Rating Scale [47] that best resembled the following measurement reactivity variables of interest: app satisfaction, app stress, app overload, and app usefulness.

App stress was measured to control for the reactivity of participants while performing ESM with this app with the item “I felt more nervous than usual, while being vigilant to receive the App’s notifications.”

App overload was used to control for whether the length of the ESM assessments (ie, self-reported surveys and trials of the app-based cognitive task) was generating overload in participants: “The number of exercises to perform in each signal was excessive.”

We also included an item to measure whether participants found the app useful in terms of facilitated introspection to understand their feelings, cognitions, and behaviors (app usefulness) with the item “Using the App has helped me to be more conscious about my emotional and cognitive responses through the day and across days.”

Global satisfaction with the app was assessed using a Likert scale ranging from 0 to 10, with 10 being highly satisfied: “Indicate, from 0 to 10, your overall satisfaction with the app.”

Finally, we used the System Usability Scale (SUS) [48] to measure the usability of the new ESM tool. Usability was assessed at the end of the study to estimate whether the new software had been experienced as usable. This scale has been previously validated [49], and it measures aspects such as complexity, technical support, integration, consistency, and general satisfaction. We computed the overall score following the indications of the author, which ranged from 0 to 100, with scores >68 being considered above average.

##### App Compliance

Compliance was defined in terms of two different dependent variables: latency and missing responses. Latency was indexed by the time participants took to respond to each signal (ie, the time lag between the prompt and the actual response, which had a maximum of 60 minutes). Missing responses were indexed using scheduled ESM prompts that were not completed by the participant.

#### Analytic Plan

We first conducted descriptive analyses of the data and used correlation analyses to study the relationships between participants’ symptoms (depression and anxiety levels), usability scores, and variables related to measurement reactivity (app stress, app overload, app usefulness, and satisfaction).

Then, to test factors accounting for individual differences in compliance, we ran multilevel analyses taking into account the nested structure of the data because of the repeated measures design (ie, observations nested within days and days nested within persons). This permitted the examination of the momentary variation of compliance variables (latency and missing responses) across prompts, considering the variability in intrapersonal and interpersonal variables. We used the *lme4* R package [50] to conduct the models predicting latency responses and missing responses. We applied the function *glmer* for latency because of the reaction time characteristics [51], specifying the family as Gamma. The *glmer* function was also used in models predicting missing responses, as this variable was coded as categorical, specifying family as a binomial. In all the models, we specified crossed random effects at the individual level. All models were fit by maximum likelihood estimation.

We first modeled an empty model, with each compliance variable predicted by its intercept. After that, we added one predictor variable at a time (univariate models) and then fitted a model with all predictors included simultaneously (multivariable model) to test whether the effects of predictor variables changed once the remaining variables were included. Fixed slopes were specified for all the models.

Thus, the time of day was entered as a level 1 predictor and day as a level 2 predictor. To explore whether individual differences in depression and anxiety levels affected compliance during the ESM study, these variables were introduced as level 3 predictors. Variables such as app stress, app overload, app usefulness, global satisfaction with the app, and usability were also introduced as level 3 predictors. We performed grand centering transformation of all the variables introduced as level 3 predictors.

#### Ethics Approval

The study was approved by the Faculty Ethical Committee of Complutense University of Madrid (Protocol Code Ref. 2019/20-028) and complied with the Declaration of Helsinki’s ethics standards.

### Results

#### Sample Characteristics and General App Performance

Initially, we recruited 102 university students. The level of abandonment was low; only 8.8% (9/102) of participants stopped responding to the ESMs and did not finish the study. Of those 9 participants, 5 (56%) missed the last 5 ESM prompts, and 4 (44%) responded only once. In addition, 8.8% (9/102) participants did not respond to the last questionnaire after completing the ESM protocol. Therefore, of the 102 participants, the final sample comprised 84 (82.4%) participants, with a mean age of 20.03 (SD 2.19) years, ranging from 18 to 29 years. We found a low mean number of missing responses per participant—1.43 (SD 1.97), ranging from 0 to 8 missing responses per participant. The mean levels of latency per participant found in this study were 16.38 (SD 7.37), ranging from a minimum of 3.24 minutes to a maximum of 34.37 minutes per participant. As shown in Table 1, participants presented mild levels of depression and anxiety, as measured with the CES-D and GAD-7, respectively. Mean scores for measurement reactivity showed moderately high levels of stress related to the use of the app. The mean app overload showed medium levels, pointing that the number of measures per assessment (questionnaires and cognitive bias tasks) was not burdensome. The extent to which participants found the app useful reflected moderately high scores, whereas their general satisfaction with the app showed medium levels.

**Table 1:** Sample characteristics and general app performance (study 1; N=84).

Characteristics	Study 1 sample
Sex (female), n (%)	75 (89)
Age (years), mean (SD)	20.03 (2.19)
Anxiety (GAD-7^a^; 0-21), mean (SD)	5.55 (3.82)
Depression (CES-D^b^ 8; 0-24), mean (SD)	7.64 (4.07)
App stress (1-5), mean (SD)	3.38 (1.50)
App overload (1-5), mean (SD)	2.69 (1,24)
App usefulness (1-5), mean (SD)	3.18 (1.30)
Satisfaction with the app (1-10), mean (SD)	6.95 (1.61)
Usability (SUS^c^; 0-100), mean (SD)	81.01 (12.08)

^a^GAD-7: Generalized Anxiety Disorder-7.

^b^CES-D: Center for Epidemiologic Studies–Depression.

^c^SUS: System Usability Scale.

Importantly, participants reported high levels of usability measured by the SUS.

Out of 100, it reached a mean usability score of 81.01 (SD 12.08), reflecting its ease of use and learnability. According to the authors who validated the scale [49], scores >70 are considered above average and acceptable, and >80.30 is in 10% of the best-rated systems [49].

#### Correlation Analyses

Correlation analyses were conducted to test how the levels of emotional psychopathology (ie, depression and anxiety levels) are related to measurement reactivity and usability indices. Given the nonnormal distribution of these variables, we conducted Spearman correlation analyses, which are shown in Table S1 in Multimedia Appendix 1. First, we found a positive, significant relationship between depression and anxiety levels (*r=*0.67; *P*<.001), indicating a relatively high degree of comorbidity among both types of symptoms. Nonetheless, neither anxiety nor depression levels were related to individual differences in the variables of user experience with the app or its usability, suggesting that such ratings were not affected by individual differences in symptom levels. Furthermore, we found significant correlations between usability and most of the measurement reactivity variables. Usability was positively related to app usefulness and satisfaction (*r*=0.31, *P*=.004 and *r=*0.60, *P*<.001, respectively) and negatively related to app overload (*r*=−0.46; *P*<.001) but not to app stress (*r*=−0.21; *P*=.05). These results show the importance of focusing on user experience when developing new eHealth app-based assessment methods, as it seems to be closely related to measurement reactivity and, in turn, the methodological quality of the design. In addition, app overload was significantly positively related to app stress (*r*=0.23; *P*=.02) and negatively related to satisfaction with the app (*r*=−0.30; *P*=.008), indicating that feeling burdensome because of the length of evaluations is related to the stress generated by the notifications’ requirements and to lower satisfaction with the app.

#### Multilevel Analyses

##### Latency

We conducted a series of transformations to control for the distribution of the outliers. Outliers were substituted with the upper or lower threshold of each participant based on the IQR. After that, we calculated the interclass correlation coefficient (ICC) for the empty model, showing a value of 14% for between-person variance. After that, we performed linear mixed models to test the predictors of the variability of latency when responding to experience sampling.

First, we conducted a series of univariate models, including 1 predictor at a time, and then tested them in a multivariate model to determine whether those effects remained significant after the inclusion of all predictor variables simultaneously. This information can be found in Table 2.

Analyses testing the effects of time-related variables on latency showed a significant effect of the day on responses’ latency in the univariate model (estimate 1.03 [SE 0.32]; *P*<.001), which remained significant in the multivariable model (estimate 1.00 [SE 0.22]; *P*<.001), indicating that a change of 1 unit on the score scale of the day (ie, 1 day passed) produced a 1.00 point of increase in latency. This indicated that as the study progressed, response latencies were longer. Furthermore, we did not find a significant effect of time of the day, neither in the univariate model nor in the multivariate model. Analyses testing the effects of emotional symptomatology on latency showed no significant effects of depression or anxiety levels when introduced as single predictors or in the multivariate model. Thus, participants’ levels of symptomatology did not influence their response latencies. Analyses testing the relationship between app-related variables and latency rates showed no significant effects of any of the variables when introduced as single predictors in the univariate models. In the multivariate model, a significant effect of app usefulness was found (estimate 1.38 [SE 0.61]; *P*=.02), indicating that participants who found the app as more useful took more time to respond to the notifications (an increase of 1 point in the score scale of app usefulness was related with an increase of 1.38 points in latency).

**Table 2:** Predictors of response latency (study 1).

Outcome variable: latency			Estimate (SE)		*t* value	*P* value
**Empty model**						
	Intercept	15.91 (0.74)		21.42		<.001
**Univariate models**						
	Day	1.03 (0.22)		4.70		<.001
	Time of day	−0.60 (0.37)		−1.61		.11
	Depression	0.11 (0.19)		0.59		.55
	Anxiety	−0.05 (0.20)		−0.26		.80
	App stress	−0.63 (0.50)		−1.26		.21
	App overload	0.14 (0.61)		0.24		.81
	App usefulness	0.96 (0.56)		1.72		.09
	App satisfaction	−0.30 (0.46)		−0.64		.52
	Usability	0.02 (0.06)		0.41		.69
**Multivariate model**						
	Day	0.10 (0.22)		4.58		<.001
	Time of day	−0.53 (0.36)		−1.46		.15
	Anxiety	−0.36 (0.27)		−1.31		.19
	Depression	0.40 (0.26)		1.55		.12
	App stress	−0.43 (0.50)		−0.86		.39
	App overload	0.27 (0.64)		0.42		.68
	App usefulness	1.38 (0.61)		2.28		.02
	Satisfaction	−1.09 (0.60)		−1.81		.07
	Usability	0.05 (0.08)		0.60		.55

##### Missing

The ICC calculation of the empty model predicting missing responses showed a value of 41% for between-person variance. Analyses testing the effect of time-related variables on missing responses showed significant effects of the day number both in the univariate model (estimate 0.23 [SE 0.08]; *P*=.004) and in the multivariable model (estimate 0.24 [SE 0.08]; *P*=.003). Time of the day did not show a significant effect on missing responses in the univariate or multivariate models. This information can be found in Table 3. Therefore, as the study progressed, the number of missing responses became higher, whereas the time of the day in which notifications were sent did not show an effect on missing responses. Analyses testing the effects of emotional symptomatology on missing responses showed no significant effects of depression or anxiety levels when introduced as single predictors or when included in the multivariate model. Finally, analyses testing the relationship between app-related variables and missing response rates showed no significant effects of app stress, app usefulness, app overload, satisfaction, or usability on missing responses. These effects were not significant in either the univariate or the multivariate models. Therefore, we can assume that technical, personal, or usability-related variables were not related to the differential missing response rates.

**Table 3:** Predictors of missing responses (study 1).

Outcome variable: missing		Estimate (SE)	*z* value	*P* value
**Empty model**				
	Intercept	−3.14 (0.29)	−10.7	<.001
**Univariate models**				
	Day	0.23 (0.08)	2.91	.004
	Time of day	0.15 (0.13)	1.16	.25
	Anxiety	0.01 (0.06)	0.2	.84
	Depression	0.02 (0.05)	0.37	.71
	App stress	−0.22 (0.15)	−1.51	.13
	App overload	−0.27 (0.17)	−1.55	.12
	App usefulness	−0.13 (0.17)	−0.81	.42
	App satisfaction	−0.09 (0.14)	−0.68	.50
	Usability	0.02 (0.02)	0.91	.37
**Multivariate model**				
	Day	0.24 (0.08)	3.06	.002
	Time of day	0.18 (0.13)	1.33	.18
	Anxiety	0.03 (0.08)	0.37	.71
	Depression	−0.01 (0.08)	−0.15	.88
	App stress	−0.19 (0.15)	−1.26	.21
	App overload	−0.28 (0.20)	−1.38	.17
	App usefulness	−0.09 (0.19)	−0.50	.64
	App satisfaction	−0.31 (0.19)	−1.62	.11
	Usability	0.03 (0.03)	1.12	.26

### Discussion (Study 1)

The main aim of this study was to examine the usability and feasibility (ie, compliant use) of a new ESM tool that integrates self-reported assessments of affective experience and a cognitive task assessing attention and interpretation biases. A series of multiple time, person, and system reactivity variables were tested as potential predictors of compliance. First, participants reported high levels of usability for the new ESM system, with scores on 10% of the best-rated systems, according to the SUS criteria. Compliance was also high in terms of both low latencies and missing responses. As for compliance predictors, we found significant effects of the day number on response latency and missing responses, indicating that latencies became longer, and there were higher missing rates as the study progressed. These results are in line with previous literature, which supports that lower compliance is found as the days of the study progress [35].

Individual differences in depression and anxiety levels did not act as significant predictors of compliance for either latency or missing responses. These results suggest that adequate compliance with the ESM system is not affected by participants’ subclinical depression and anxiety levels, which is a crucial aspect of the aim of this type of new ESM system. To advance future clinical implementation, further research should analyze these issues in participants presenting with higher levels of depression and anxiety symptomatology to test the effect of clinical status on compliance with this ESM system integrating both self-reports and cognitive tasks [36].

Furthermore, system-related variables were not significantly associated with compliance rates, except app usefulness. Interestingly, participants who found the app to be more useful were those who were slower in responding to momentary notifications. This might be indicative of an attempt to find the more proper moments to adequately perform the app tests and assessments within the allowed 1-hour period after notification. Nonetheless, this effect was only evident in the multivariate model, whereas the univariate analyses did not show any direct association and should be considered cautiously until further replication.

Overall, the results showed high usability and feasibility for the use of the new ESM system, with few factors substantially accounting for its adequate compliance. Nonetheless, we should note various limitations in this study. First, we used single items to evaluate variables related to measurement reactivity, such as app stress, overload, usefulness, and satisfaction, which might at least partly obscure some of their potential associations with compliance.

Furthermore, there might be certain limitations in the generalizability of the results, given the relatively short number of days evaluated (ie, 5 days). These current findings invite further replication using the new ESM system in protocols with more extensive durations that are comparable with other ESM research considering compliance predictors. For these reasons, we performed a second ESM study with a longer protocol (ie, 10 days of assessments), integrating the study of measurement reactivity variables not only through single items but also through different validated scales.

Thus, study 2 overcame the abovementioned issues, adding further contributions to knowledge in particular areas. First, in study 2, we expanded the sample of participants and extended the duration of the study. It is important to verify whether the results, in terms of reactivity with the app and compliance, would change when participants were evaluated twice as long. In addition, we performed a significant methodological improvement by including validated subscales to measure the variables of app-related stress, overload, usefulness, and satisfaction to derive precise knowledge of their relationship with compliance rates.

## Study 2

### Methods

#### Participants

A sample of 135 undergraduate students (age: mean 20.52, SD 2.31 years) was recruited from the Faculty of Psychology at Complutense University of Madrid between April and May 2021. The participants received extra credit for participating in the study.

#### Procedure

Participants individually attended an app-based introductory session in which they received information about the study. They were trained to use the app in which the ESM system was integrated and performed a practice exercise of self-report measures and cognitive tasks. On the first day, they also completed the informed consent and a baseline questionnaire assessing demographic variables, measures of depression and anxiety levels, and other scales not relevant to the aim of this study. One day after the introductory session, participants were instructed to start completing the ESM protocol through the app on their phones each time they received a survey notification. A systematic sampling approach was used to determine the random signaling schedules. Experience sampling assessments were programmed to be sent to participants 3 times a day for 10 days. These assessments were prompted randomly between 10 AM and 9 PM at three time intervals (10 AM to 11 AM, 3 PM to 4 PM, and 8 PM to 9 PM). Participants had 1 hour since the time they received the notification to complete the assessment. At each assessment, they completed assessments of stress, current affect, and emotion regulation strategies and performed the cognitive bias task exactly as in study 1. Furthermore, as in study 1, compliance-related information, such as latency of response and missing and abandonment rates, was gathered. At the end of the study, participants completed a brief questionnaire that accounted for variables related to measurement reactivity and user experience. Questionnaires were completed at baseline and at the end of the study using Qualtrics software.

#### Instruments

##### Baseline Assessments

Depression and anxiety symptom levels were measured using the CES-D-8 [41] and the GAD-7 [42], respectively, as in study 1. The internal consistencies for the CES-D 8 and GAD-7 in this study were *α*=.88 and *α*=.86, respectively.

##### Momentary Assessments

As in study 1, assessments referring to self-reports of perceived ongoing stress, use of emotion regulation strategies, and current mood states were evaluated through app-based self-reports. In each signal, participants also completed a cognitive task assessing momentary attention and interpretation biases, which was based on the SST [17]. They were required to complete 15 phrases at each beep for a total of 3 times a day for 10 days. Further details of all assessments are provided in Multimedia Appendix 1.

##### Assessments at the End of the Study

As in study 1, after completing the ESM procedure, participants received the last questionnaire survey, which measured system usability, measurement reactivity, and user experience using the same assessments. Furthermore, study 2 also included the assessment of measurement reactivity and user experience dimensions using additional subscales extracted from validated questionnaires to measure stress, overload, usefulness, and satisfaction related to the use of the app.

Usability was measured using the SUS [48], as in study 1. The internal consistency of this scale in the study was *α*=.75.

Stress reactivity resulting from app use (subscale) was assessed through the Pressure/Tension subscale of the Intrinsic Motivation Inventory [52]. This subscale contains 5 items that measure the negative reactivity of participants while performing ESM with the tool. The internal consistency of this scale in the study was *α*=.56.

Overload resulting from app use (subscale) assessed the experienced negative affect and the degree of control and effort required during the completion of the ESM protocol. This was assessed using the Perceived Usability subscale from the User Engagement Scale [53], which comprises 8 items. The internal consistency of this scale in the study was *α*=.84.

To measure app usefulness (subscale), we used the Value subscale of the Intrinsic Motivation Inventory [52] to assess how participants found it useful to complete the ESM protocol by being more conscious of their own emotional and cognitive states. The internal consistency of this scale in the study was *α*=.94.

Satisfaction with the app was assessed using the Endurability subscale from the User Engagement Scale [53], referred to as the overall success of the interaction and users’ willingness to recommend the app to others or engage with it in the future. The internal consistency of this scale in the study was *α*=.90.

##### App Compliance

Participants’ level of commitment was also assessed in study 2. Therefore, latency and missing responses were recorded to perform compliance analyses, exactly as in study 1.

#### Analytic Plan

The analytic plan in study 1 was entirely reapplied in study 2. We conducted a descriptive analysis and performed Spearman correlation analyses because of the nonnormal distribution of the variables of interest. In addition, similar mixed model regression analyses were performed, as in study 1. Furthermore, we conducted an additional multivariate model in which we included methodological variables (day and time of the day), emotional symptomatology, usability, and variables related to the use and reactivity to the ESM system, as measured through the further included validated subscales (stress, overload, usefulness, and satisfaction). Therefore, separate analyses were conducted considering the item-based indices of measurement reactivity (ie, for the replication of the study 1 results) and further scale-based indices of measurement reactivity included in this study.

#### Ethics Approval

The study was approved by the Faculty Ethical Committee of Complutense University of Madrid (Protocol Code Ref. 2020/21-023) and complied with the Declaration of Helsinki’s ethics standards.

### Results

#### Sample Characteristics and General App Performance

As shown in Table 4, participants presented moderate depression and low anxiety levels, as measured with the CES-D 8 and GAD-7, respectively. Initially, we recruited 139 participants, from whom we found high compliance, as the level of abandonment was very low—2 (1.46%) participants in the sample (corresponding to 2 participants who completed <6 assessments). These 2 participants were excluded from the analysis because of the noncompletion of the questionnaires on usability and measurement reactivity at the end of the ESM protocol. Therefore, of the 139 participants, the final sample included in the analyses comprised 135 (97.1%) participants with a mean age of 20.51 (SD 2.31) years. We found a low mean number of missing responses per participant—3.60 (SD 3.32), ranging from 0 to 17 missing responses per participant. The mean levels of latency per participant found in this study were 23 to 79 (SD 8.1) minutes, ranging from a minimum of 6.15 minutes to a maximum of 49.64 minutes per participant.

**Table 4:** Sample characteristics and general app performance (study 2; N=135).

Characteristics	Values
Sex (female), n (%)	116 (85.9)
Age (years), mean (SD)	20.51 (2.31)
Anxiety (GAD-7^a^; 0-21), mean (SD)	8.38 (4.11)
Depression (CES-D^b^ 8; 0-24), mean (SD)	9.09 (4.89)
App stress (item; 1-5), mean (SD)	2.96 (1.30)
App overload (item; 1-5), mean (SD)	2.93 (1.25)
App usefulness (item; 1-5), mean (SD)	3.44 (1.14)
Satisfaction (item; 1-10), mean (SD)	6.62 (1.92)
App stress (IMI^c^ subscale; 1-7), mean (SD)	3.09 (1.09)
App overload (UES^d^ subscale; 1-5), mean (SD)	1.86 (0.77)
App usefulness (IMI subscale; 1-7), mean (SD)	4.16 (1.37)
Satisfaction subscale (UES subscale; 1-5), mean (SD)	3.40 (0.95)
Usability (SUS^e^; 0-100), mean (SD)	82.17 (11.74)

^a^GAD-7: Generalized Anxiety Disorder-7.

^b^CES-D: Center for Epidemiologic Studies–Depression.

^c^IMI: Intrinsic Motivation Inventory.

^d^UES: User Engagement Scale.

^e^SUS: System Usability Scale.

In general, participants showed medium levels in variables related to the negative reactivity of the app (stress and overload), both measured using single items and their corresponding subscales. Participants showed medium to high scores in perceived usefulness when using the app and global satisfaction, as reflected by both the single items and the corresponding subscales. Overall, the results showed that participants did not report feeling stressed or overloaded because of the use of the system while participating in the study. Furthermore, participants found the app useful for its purpose of facilitating awareness of internal affective and cognitive states.

In addition, importantly, the usability of the app was rated as very high (mean 82.17, SD 11.74), as measured by SUS, where scores >80.30 are considered to be in 10% of the best-rated systems.

#### Correlation Analyses

The set of Spearman correlations is shown in Table S2 in Multimedia Appendix 1. As in study 1, we found a significant relationship between anxiety and depression levels (*r*=0.71; *P<*.001), indicating a certain degree of comorbidity among the symptoms. Second, we found a significant positive relationship between anxiety levels and reactive stress (measured by a single item; *r*=0.21; *P*=.01), indicating that participants who scored higher on anxiety levels felt more nervous or stressed because of ESM notifications. However, these results were not replicated in relation to the app stress subscale.

As in study 1, usability was significantly related to various variables concerning the reactivity with the app. We found negative significant relationships between usability and app stress (single item *r*=−0.21, *P*=.02; subscale *r*=−0.39, *P*<.001) and between usability and app overload (single item *r*=−0.40, *P*<.001; subscale *r*=−0.47, *P*<.001). We also found positive significant relationship between usability and app usefulness (single item *r*=0.26, *P*=.002; subscale *r*=0.43, *P*<.001) and app satisfaction (single item *r*=0.50, *P*<.001; subscale *r*=0.43, *P*<.001). Therefore, participants who found the app more usable showed lower levels of app stress and overload and higher levels of app usefulness and satisfaction with its use.

Variables referring to the negative measurement reactivity (stress and overload) were significantly and positively correlated (between single items*: r*=0.34, *P*<.001; between subscales: *r*=0.57, *P*<.001), indicating that those participants who felt more stressed because of notification requirements also felt higher overload because of completing each assessment. On the other hand, we found positive and significant correlations between general satisfaction with the app and perceived usefulness (between single items: *r*=0.45, *P*<.001; between subscales: *r*=0.72, *P*<.001). Overall, these results replicate and extend the previous findings in study 1 and are in line with previous research validating app-based tools of psychological assessment [54].

#### Multilevel Analyses

##### Latency

As in study 1, we conducted a series of transformations to control for the distribution of outliers. After that, we used linear mixed models, fitted by maximum likelihood estimation, to test time-, person-, and system-related predictors of the variability of latency when responding to experience sampling (results of empty univariate and multivariate models predicting latency can be found in Table 5). We used the same models as in study 1, and an additional multivariate model was tested, with variables related to measurement reactivity measured through the corresponding subscales. We also calculated the ICC for the empty model predicting latency responses, which showed a value of 22% for between-person variance.

Analyses testing the effects of design and time-related variables on latency showed a significant effect of the day number, showing the same effect in both the univariate (estimate 0.28 [SE 0.09]; *P*=.001) and multivariate models (estimate 0.29 [SE 0.09], *P*=.01; estimate 0.28 [SE 0.09], *P*=.001, respectively). Time of the day also showed significant effects on latency responses in the univariate model (estimate −0.42 [SE 0.21]; *P*=.04) and in both multivariate models (estimate −0.44 [SE 0.21], *P*=.04; estimate −0.43 [SE 0.21], *P*=.04, respectively). The results partially replicated those from study 1, showing that response latencies became longer as the study progressed; however, only in study 2, later notifications within the day were further related to lower latency rates and, therefore, to faster responses in those moments of the day.

As in study 1, analyses testing the effects of symptomatology levels on latency showed no significant effects of depression and anxiety on the latency response rates. Therefore, the levels of emotional symptomatology of the participants did not affect their latencies to respond to notifications.

Finally, analyses testing the effects of app-related variables on latency showed a significant effect of app overload, measured through the subscale (estimate 1.52 [SE 0.71]; *P*=.03) in the univariate model. This effect remained significant in the multivariate model when introduced with the remaining variables (estimate 2.20 [SE 1.02]; *P*=.03). Thus, higher experienced overload during the ESM protocol was associated with longer response latencies to notifications.

**Table 5:** Predictors of response latency (study 2).

Outcome variable: latency		Estimate (SE)	*t* value	*P* value
**Empty model**				
	Intercept	23.76 (0.50)	47.9	<.001
**Univariate models**				
	Day	0.28 (0.09)	3.22	.001
	Time of day	−0.42 (0.21)	−2.04	.04
	Anxiety	0.16 (0.12)	1.34	.18
	Depression	−0.03 (0.10)	−0.33	.73
	App stress item	0.36 (0.38)	0.95	.35
	App overload item	0.54 (0.40)	1.37	.17
	App usefulness item	−0.04 (0.43)	−0.08	.94
	App satisfaction item	–0.313 (0.26)	−1.25	.21
	App stress subscale	0.57 (0.47)	1.21	.23
	App overload subscale	1.56 (0.66)	2.37	.02
	App usefulness subscale	0.26 (0.37)	0.70	.48
	App satisfaction subscale	0.05 (0.53)	0.10	.92
	Usability	−0.01 (0.04)	−0.21	.83
**Multivariate model 1 (item variables)**				
	Day	0.29 (0.09)	3.31	<.001
	Time of day	−0.44 (0.21)	−2.1	.04
	Anxiety	0.33 (0.17)	1.94	.05
	Depression	−0.23 (0.14)	−1.59	.11
	App stress item	0.05 (0.45)	0.11	.91
	App overload item	0.34 (0.49)	0.70	.48
	App usefulness item	0.20 (0.52)	0.39	.70
	App satisfaction item	−0.39 (0.38)	−1.024	.31
	Usability	0.04 (0.05)	0.71	.48
**Multivariate model 2 (subscale variables)**				
	Day	0.28 (0.09)	3.25	.001
	Time of day	−0.43 (0.21)	−2.07	.004
	Anxiety	0.24 (0.17)	1.44	.15
	Depression	−0.16 (0.14)	−1.16	.24
	App stress subscale	−0.02 (0.59)	−0.03	.98
	App overload subscale	2.39 (0.96)	2.50	.01
	App usefulness subscale	0.10 (0.56)	0.18	.86
	App satisfaction subscale	0.51 (0.83)	0.61	.54
	Usability	0.06 (0.06)	0.99	.32

##### Missing

First, we calculated the empty model predicting missing responses and the ICC, which showed a value of 20% for between-person variance. Analyses testing the effects of design time–related variables on missing responses showed that time of the day was a significant predictor in both the univariate model (estimate 0.17 [SE 0.02]; *P*=.01) and the multivariate models 1 and 2 (estimate 0.16 [SE 0.06]; *P*=.01).

As for the analyses of the effects of emotional symptomatology on missing response rates, nonsignificant effects of depression and anxiety levels were found on missing responses.

Finally, analyses testing the relationship between app-related variables and missing response rates showed that item-based app stress had significant effects in the univariate model (estimate 0.21 [SE 0.07]; *P*=.004), which remained significant in multivariate model 1 (estimate 0.18 [SE 0.08]; *P*=.004). Item-based overload also showed a significant effect in the univariate model (estimate 0.16 [SE 0.08]; *P*=.04); however, this effect did not remain significant in multivariate model 1 (estimate 0.08 [SE 0.09]; *P*=.36). As for subscale-based measurements (model 2), app overload measured through the subscale also showed significant effects on missing rates when introduced as a single predictor (estimate 0.29 [SE 0.12]; *P*=.02), as well as on app satisfaction measured through a subscale (estimate –0.20 [SE 0.10]; *P*=.049). However, when all predictors were introduced in multivariate model 2, neither of these variables showed any significant effect (results of empty univariate and multivariate models predicting missing responses can be found in Table 6).

**Table 6:** Predictors of missing responses (study 2).

Outcome variable: missing		Estimate (SE)	*z* value	*P* value
**Empty model**				
	Intercept	−2.43 (0.11)	−20.73	<.001
**Univariate models**				
	Day	0.01 (0.02)	0.39	.70
	Time of day	0.17 (0.02)	2.53	.01
	Anxiety	0.03 (0.02)	1.39	.17
	Depression	0.005 (0.02)	0.23	.82
	App stress item	0.21 (0.07)	2.90	.004
	App overload item	0.16 (0.08)	2.08	.04
	App usefulness item	−0.06 (0.09)	−0.72	.47
	App satisfaction item	−0.07 (0.05)	−1.45	.15
	App stress subscale	0.10 (0.09)	1.14	.25
	App overload subscale	0.29 (0.12)	2.37	.02
	App usefulness subscale	−0.10 (0.07)	−1.42	.16
	App satisfaction subscale	−0.20 (0.10)	−2.01	.04
	Usability	−0.01 (0.01)	−1.48	.14
**Multivariate model 1 (item variables)**				
	Day	0.01 (0.02)	0.41	.68
	Time of day	0.16 (0.06)	2.54	.01
	Anxiety	0.04 (0.03)	1.15	.25
	Depression	−0.03 (0.03)	−1.01	.31
	App stress item	0.18 (0.08)	2.11	.04
	App overload item	0.08 (0.09)	0.91	.36
	App usefulness item	−0.004 (0.10)	−0.04	.97
	App satisfaction item	0.02 (0.07)	0.31	.76
	Usability	−0.01 (0.01)	−0.50	.62
**Multivariate model 2 (subscale variables)**				
	Day	0.01 (0.02)	0.39	.70
	Time of day	0.16 (0.06)	2.55	.01
	Anxiety	0.05 (0.03)	1.42	.16
	Depression	−0.02 (0.03)	−0.84	.40
	App stress subscale	−0.02 (0.11)	−0.20	.84
	App overload subscale	0.21 (0.18)	1.17	.24
	App usefulness subscale	−0.01 (0.10)	−0.14	.90
	App satisfaction subscale	0.11 (0.16)	−0.71	.48
	Usability	0.001 (0.01)	0.11	.91

### Discussion (Study 2)

Study 2 was conducted to test the new ESM system using a longer ESM protocol than in study 1 and increase the methodological quality of app reactivity measurement from study 1, adding further assessments of these characteristics through validated subscales of app stress, overload, usefulness, and satisfaction.

Study 2 replicated the high usability scores of the new ESM system, with high levels over the 90th percentile of the SUS scale. Higher usability was related to several indicators such as lower user stress and overload and higher usefulness and satisfaction, replicating and extending the findings from study 1 on the acceptability of the novel procedure.

Furthermore, compliance with the protocol was high, as in study 1, showing that extending the protocol regime did not affect general compliance. After performing mixed model analyses, we replicated the findings from study 1 on the time-related predictors of compliance with the procedure. We found a significant effect of day number on response latency, indicating that latencies became longer as the study progressed. These results are in line with the findings of study 1. In addition, a negative relationship between the time of the day and latency was found, indicating that in earlier notifications, latencies tended to be slower. This result was not found in study 1 and may indicate that extending the duration of the ESM protocol may permit the identification of performance patterns that may remain undetected for shorter durations (ie, a 5-day duration in study 1). Furthermore, we found that time of the day was also a significant predictor of missing responses, indicating more missing responses in later notifications of the day (interval between 8 PM and 9 PM). These results are in line with previous research showing lower compliance in terms of missing response rates in later daily notifications [28].

In addition, as in study 1, depression and anxiety levels were not found to significantly predict variability with ESM compliance, which is important for the future implementation of these measurements in clinical samples. Importantly, study 2 was completed from April to May 2021, when the COVID-19 pandemic was a persistent source of threat, and this was evidenced by participants’ symptom levels, which were higher than those for participants in study 1, which was completed in 2019. Thus, although the samples were comparable in terms of sociodemographic characteristics, depression and anxiety reached higher overall subclinical levels in the sample of study 2. The fact that symptom levels in study 2 did not affect levels of compliance is clearly indicative of the feasibility of the ESM system for further implementation in clinical settings in the future.

In terms of measurement reactivity, we found significant effects of the measurement reactivity variables in predicting compliance in study 2. Higher app overload was related to more response latencies, whereas app stress significantly predicted more missing responses. These results are in line with previous research suggesting that a higher negative measurement reactivity is related to lower compliance rates [23]. Interestingly, these effects were not found in study 1, suggesting that the influence of these variables on the compliant performance of the new ESM system may only emerge when using more extensive protocols of assessment (ie, 10 days vs 5 days).

## Discussion

### Principal Findings

The aim of this study was to investigate the usability and feasibility of using a new ESM system, which integrates self-report questionnaires and an app-based cognitive task that allows the assessment of ecological indicators of cognitive-affective mechanisms implicated in emotion regulation and emotional symptoms, through 2 differently extensive ESM designs (studies 1 and 2). Study 1 required participants to respond to 3 assessments per day for 5 days. Study 2 integrated the ESM design into a protocol of assessments of 3 times a day for 10 days.

Across studies, we found similar results in terms of the average levels of usability of the novel ESM system, indicating its ease of use and learnability. This is particularly relevant because of the development of a new system that integrates an app-based cognitive task into the ESM procedure. Previous platforms have been developed to repeatedly assess affect through ESM, provide a visual environment [55-57], and highlight the importance of usability testing. In the case of the new ESM system used in our studies, the results on usability showed a very high level of learnability (ie, over the 90th percentile of the SUS), which reflects the ease of use and acceptability by the participants. In fact, we found that usability rates were significantly related to lower negative measurement reactivity (stress and overload) and more perceived usefulness and satisfaction with the app in both studies. Therefore, the use of this ESM system does not require an advanced technical background, and its implementation would be appropriate in the general population. The mean values of reactivity to the assessment measure were also similar across both studies, such that the levels of app overload were moderate (burdensome because of the length of assessments) and app usefulness was high (utility to increase users’ conscientiousness of their internal psychological states). Furthermore, person-related variables of depression and anxiety did not affect compliance with the ESM protocol. This also informs on the feasibility of further expanding and implementing the new ESM system in clinical settings.

Moreover, we found different results between studies, such that only in study 2 (ie, with a more extensive protocol of 10 days), individual differences in app overload and stress accounted for lower compliance rates. However, it must be noted that the degree of between-person variability in compliance rates was relatively low (as indicated by the ICC derived from empty models), suggesting that such effects might turn out anecdotical.

### Limitations

Despite the implications of these studies, some limitations must be considered. First, study 2 was conducted during the COVID-19 pandemic, which makes it difficult to generalize the results in terms of compliance with other regular contexts of evaluation, given the exceptional circumstances in which the participants found themselves at that time. Despite this, measures of reactivity to the mobile app and its usability remained very similar between both studies, which indicates that they may depend more on other design- or sample-related characteristics rather than on contextual conditions. This implies the feasibility of applying similar designs with the new ESM system in different contexts in a reliable manner, including the use of the novel app for the study of cognitive and emotional dynamics within individuals across multiple contexts across time. Furthermore, in terms of between-person differences, the relatively high scores in depression and anxiety levels in the sample in study 2 (subclinical levels) minimally affected compliance with the app, which is indicative of its feasibility for further implementation with other types of at-risk and clinical populations.

Second, studies 1 and 2 differ in the way the ESM system was implemented, as it was used through a mobile app in the former one, whereas in the latter study, an integrated app system that could be completed both on phones and computers was used. Importantly, despite this difference, participants showed similar scores on usability and measurement reactivity with the system, pointing to the adequacy of using either format of the new ESM system, depending on specific user requirements.

Third, measurement reactivity variables were only measured through item-based assessments in study 1. This was solved in study 2, in which we added further validated subscales to measure the reactivity variables. Although these subscales resembled the constructs gathered by the single items, the results were not fully replicated in multivariable models 1 (item-based) and 2 (scale-based) of study 2. As these scales showed high reliability in general and added higher methodological quality, it is recommended to use them to replicate results in future studies.

Future research should also focus on incorporating other cognitive evaluation tasks into ESM systems, not just self-report assessments, combining both implicit and explicit assessments of emotional, behavioral, and cognitive processes. This promises to bring great wealth to the understanding of the psychological dynamic underlying the ecological mechanisms of people’s emotional dysfunctions (ie, depression and anxiety) in daily life.

### Conclusions

This study supports the validity and feasibility of the presented new ESM system. Furthermore, our findings indicate that more systematic investigations into the design characteristics influencing data quality and quantity in ESM studies are needed. The variability of compliance rates, in terms of latency and missing responses, depended on variables related to the design of the ESM procedures and measurement reactivity variables in our studies. Lower compliance was found across both studies as the days in each study progressed, and measurement reactivity variables were found to be related to lower compliance rates (higher latency and missing responses) in more extensive ESM protocols (study 2). Importantly, participants’ levels of depressive and anxiety symptomatology did not affect compliance in our study, indicating the feasibility of using this type of new system in ESM designs for multiple populations. This will permit the evaluation of ecological, cognitive, and emotional dynamics in individuals’ daily life and a better understanding of the underlying mechanisms ultimately influencing mental health.

## Appendix

Multimedia Appendix 1 Experience sampling method assessments and correlation analyses tables.

## Abbreviations

CES-D: Center for Epidemiologic Studies–Depression
ESM: experience sampling method
GAD-7: Generalized Anxiety Disorder-7
ICC: interclass correlation coefficient
SST: Scrambled Sentence Task
SUS: System Usability Scale
