# Supplementary material for: A Novel Experience Sampling Method Tool Integrating Momentary Assessments of Cognitive Biases: Two Compliance, Usability, and Measurement Reactivity Studies
Source: JMIR Form Res. 2022 Mar 28;6(3):e32537. doi: 10.2196/32537 (PMC9002591; doi:10.2196/32537)
Supplement: Multimedia Appendix 1 [file formative_v6i3e32537_app1.docx]

# Multimedia Appendix

## ESM Assessments

### Self-reported items

Current mood: To what extent do you feel at this moment (scored from 1 -not all- to 9-very much-)

#### Positive Emotions:

- Cheerful
- Content
- Energetic
- Relaxed

#### Negative Emotions:

- Nervous
- Content
- Irritable
- Tired

#### Stress appraisals

Perceived stress intensity and stress control were continuously monitored across the full ESM period. The evaluation included two different questions to assess these appraisals in relation to stressful situations experienced since the previous assessment period, framed in accordance with previous ESM research.

##### Stress intensity

Participants rated the perceived intensity of experienced stress situations during the preceding period to each prompt using the following question: “Since the last survey, to what extent what has happened has been stressful to you?” rated on a scale from 1 (not all) to 9 (very much).

##### Stress control

Participants rated the degree to which they perceived the experienced stressor at each prompt as controllable: “To what extent were you in control of what has happened since the last survey?” rated on a scale from 1 (not all) to 9 (very much).

### Emotion regulation strategy use*.*

The online tool included the assessment of the momentary degree of use of eight strategies of emotion regulation. In every ESM survey, participants had to answer to what extent they had used each strategy in relation to the stressful situations experienced since the last survey (ie, “In response to what has happened since the last survey, to what extent…). All measures of ERS use were rated on a scale from 1 (not all) to 9 (very much).

##### Reappraisal

It was assessed using an item related to perspective taking, which was based on previous ESM research monitoring momentary cognitive reappraisal use in response to stress: “… have you looked at things from a different perspective?”.

##### Active coping

An item assessed problem-solving responses, defined as conscious attempts to change a stressful situation, or contain its consequences, namely Active Coping. This item was framed in accordance with previous ESM research assessing this strategy: “… have you tried to change something about the situation?”.

##### Avoidance

The use of avoidant coping was assessed in relation to the use of an internal mental focus avoidance (ie, Experiential avoidance), which was referred to the degree of use of suppression of thoughts related to feelings and problems resulting from ongoing stress: “… have you tried to avoid thinking about your feelings and problems?”.

##### Rumination

It was framed as a strategy consisting of thinking repeatedly on the problems or feelings that one has in each given moment, namely in response to ongoing experienced stressful experiences. Thus, the question was framed in relation to previous ESM research assessing this specific process: “… have you been dwelling on your feelings and problems?”.

##### Worry

This strategy refers to repeatedly thinking on the problems that one situation could create to the person in the future. In accordance to previous ESM research, the question used to evaluate this strategy in response to ongoing stressful experiences was “… have you been worried about things that could happen?”

##### Mental change

This item was also framed on previous ESM research monitoring other forms of momentary cognitive reappraisal use in response to stress as “…have you changed the way you were taking about the situation?”

External distraction: this strategy reflects an external mental focus avoidance (external distraction), referred to the extent to which participants tried to distract themselves from ongoing stressful situations: “… have you tried to distract yourself from what was going on?”

##### Future Planning

It was framed in accordance with problem solving models considering this as a form of intentional planning of future behaviours to prevent negative consequences of stress situations that are similar to the ones previously experienced: “… have you thought about what you could do to solve similar upcoming situations in the future?”

#### Motivational levels

The online tool included the assessment of the momentary activation of different forms of promotion and prevention motivational states, through the following items scored from 1 to 9:

##### Promotion focus item 1

“To what extent has it been important to you achieving goals or aims that were relevant in your life?”

##### Promotion focus item 2

“To what extent have you been focused in searching ways to have fun or feel good?”

##### Prevention focus item 1

“To what extent it has been important to you solving problems or getting ready to cope with potential difficulties?”

##### Prevention focus item 2

“To what extent have you been focused on searching ways to feel secure, protected or calmed?”

### Online Cognitive Task

The Scramble Sentence Task (SST) was implemented to evaluate cognitive attention and interpretation biases of participants in the study at each ESM assessment.

A total of 15 scrambled sentences, composed by 6 words (eg, “looks the future bright very dismal”) was presented to participants. The number of trials was established based on previous extensive piloting of sufficient required SST trials to obtain reliable cognitive bias indices related to stress vulnerability and depression status. Participants were instructed to mentally unscramble the sentences, as fast as possible, using only 5 out of the 6 words, to create a grammatically correct and meaningful sentence. These sentences could only be unscrambled with a negative or a positive meaning (eg, “the future looks very dismal” or “the future looks very bright”). Participants were instructed to unscramble the words into the valid sentence that first came to their mind. To control for the influence of words’ positioning, the emotional words (ie, positive or negative) were always displayed in the second and fifth position. Additionally, these positions were counterbalanced, controlling that positive and negative words were similarly allocated in the second and fifth positions across trials.

The task was completed on participants’ smartphones. Each trial started with a fixation cross in the left position of the screen to promote natural left-to-right reading patterns. Participants were asked to press the cross with their finger to start the trial. Immediately after, a reading phase started, where participants had to read and mentally unscramble the words in a limited time of 14 seconds. Using a moving window procedure, the 6 words were hidden in individual boxes. In order to read them, participants had to move their finger throughout a scroll bar below the boxes to unhide the corresponding word. Once participants moved their finger from one word to another, the previous words were hidden again. During this reading phase, the position of the finger on the screen was monitored, allowing to compute the time spent (in milliseconds) reading (attending) to each word of the scrambled sentence, and thus assessing the proportion of total time attending negative over positive words (ie, negative attention bias). After the time limit, or when participants decided (pressing a “Ready” button), the final response phase began (see Figure 1).


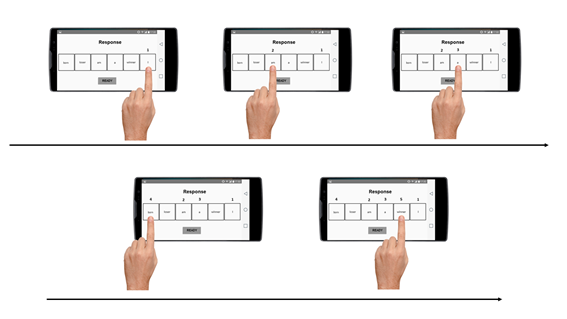
Figure 1*. Reading phase of Cognitive Task*

In the response phase, all words were then unhidden. With a time-limit of 7 seconds, participants had to create the meaningful sentence by pressing, as fast as possible and in the appropriate order, the corresponding chosen series of 5 words. If participants made any mistake during the construction of the sentence, they could modify it by unselecting the wrong word and selecting a new one (see Figure 2). Once the 5 words had been selected, participants pressed the “Ready” button at the bottom of the screen and started a new trial. The system recorded responses for each trial to compute the interpretation bias index (see below).

Figure 2*.* *Response phase of Cognitive Task*

*
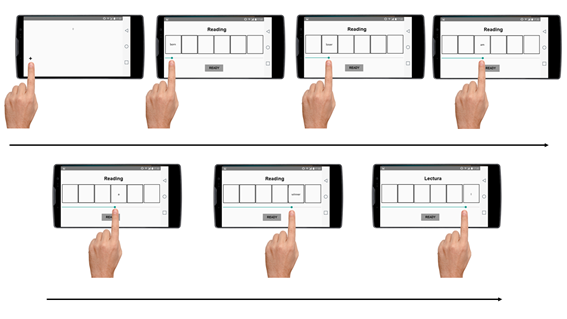
*

Attention and interpretation bias indices: The task was designed to allow for online assessment of the total time attending to negative over positive words during the reading phase as well as the proportion of negative over positive interpretations made during the response phase. The program registered the total time (in milliseconds) that participants spend reading (attending towards) negative and positive words. We analyzed the reliability of each measure. Following previous studies, an attention bias index is computed by dividing the total time attending negative words by the total time attending both emotional (ie, positive and negative) words. Therefore, values above 0.5 are indicative of an attention bias towards negative information whereas values below 0.5 are indicative of an attention bias towards positive information. The program also computed the number of positive and negative grammatically correct sentences that were unscrambled by each participant during the response phase. An interpretation bias index is computed by dividing the number of negative sentences by the total number of unscrambled sentences (ie, positive and negative) made. As with the attention bias index, values above 0.5 indicates a negative interpretation bias whereas values below 0.5 indicates a positive interpretation bias.

## Correlation analyses of Study 1 and 2

Supplementary Table 1. Correlation analyses in Study 1

| **Measures**  **Study 1**  *Spearman r* (*P* value) | **1** | **2** | **3** | **4** | **5** | **6** | **7** |
| --- | --- | --- | --- | --- | --- | --- | --- |
| **1 Depression** | 1 |  |  |  |  |  |  |
| **2 Anxiety** | .67 (<.001) | 1 |  |  |  |  |  |
| **3 Usability** | .05 (.64) | .09 (.43) | 1 |  |  |  |  |
| **4 Satisfaction** | .09 (.41) | .17 (.12) | .60(<.001) | 1 |  |  |  |
| **5 Stress** | -.09 (.44) | .02(.83) | -.21 (.052) | -.18 (.11) | 1 |  |  |
| **6 Overload** | -.16 (.16) | -.12 (.27) | -.46(<.001) | -.29(.008) | .26(.02) | 1 |  |
| **7 Usefulness** | -.03(.76) | -.11(.34) | .31 (.004) | .43(<.001) | -.10 (.35) | -.05(.66) | 1 |
|  |  |  |  |  |  |  |  |

Table 2 Supplementary. Correlation analyses in Study 2

| **Measures Study 2**  *r* (*P* value) | **1** | **2** | **3** | **4** | **5** | **6** | | **7** | **8** | **9** | **10** | **11** |
| --- | --- | --- | --- | --- | --- | --- | --- | --- | --- | --- | --- | --- |
| **1 Depression** | 1 |  |  |  |  |  | |  |  |  |  |  |
| **2 Anxiety** | .71 (<.001) | 1 |  |  |  |  | |  |  |  |  |  |
| **3 Usability** | -.09 (.31) | -.03(.72) | 1 |  |  |  | |  |  |  |  |  |
| **4 Satisfaction Item** | -.001(.91) | .017 (.85) | .50(<.001) | 1 |  |  | |  |  |  |  |  |
| **5 Stress Item** | -.17 (.047) | .21 (.014) | -.21 (.015) | -.33 (<.001) | 1 |  | |  |  |  |  |  |
| **6 Overload Item** | -.05 (.53) | .15 (.09) | -.40(<.001) | -.44(<.001) | .40 (<.001) | | 1 |  |  |  |  |  |
| **7 Usefulness Item** | -.05(.57) | -.01 (.99) | .26 (.002) | .45(<.001) | -.18 (.04) | -.15(.08) | | 1 |  |  |  |  |
| **8 Satisfaction Subscale** | -.02 (.86) | .05(.59) | .46 (<.001) | .75(<.001) | -.21(.013) | -.30(<.001) | | .53(<.001) | 1 |  |  |  |
| **9 Stress Subscale** | .14 (.12) | .07(.40) | -.39 (<.001) | -.41 (<.001) | .39(<.001) | .41(<.001) | | -.15(.08) | -.30(<.001) | 1 |  |  |
| **10 Overload Subscale** | .04 (.61) | .11(.23) | -.47 (<.001) | -.54 (<.001) | .35(<.001) | .47(<.001) | | -.20(.02) | -.40 (<.001) | .57 (<.001) | 1 |  |
| **11 Usefulness Subscale** | -.01 (.91) | .06(.58) | .43 (<.001) | .63(<.001) | -.20(.02) | -.27(.002) | | .76 (<.001) | .72 (<.001) | -.22 (.012) | -.21(.02) | 1 |
|  |  |  |  |  |  |  | |  |  |  |  |  |
|  |  |  |  |  |  |  | |  |  |  |  |  |
